# RNA Chemical Labeling with Site‐Specific, Relative Quantification by Mass Spectrometry for the Structural Study of a Neomycin‐Sensing Riboswitch Aptamer Domain

**DOI:** 10.1002/cplu.202200256

**Published:** 2022-10-11

**Authors:** Michael Palasser, Kathrin Breuker

**Affiliations:** ^1^ Institut of Organic Chemistry and Center for Molecular Biosciences Innsbruck (CMBI) University of Innsbruck Innrain 80/82 6020 Innsbruck Austria

**Keywords:** chemical probing, collisionally activated dissociation, FT-ICR, mass spectrometry, RNA

## Abstract

High‐resolution mass spectrometry was used for the label‐free, direct localization and relative quantification of CMC^+^‐modifications of a neomycin‐sensing riboswitch aptamer domain in the absence and presence of the aminoglycoside ligands neomycin B, ribostamycin, and paromomycin. The chemical probing and MS data for the free riboswitch show high exposure to solvent of the uridine nucleobases U7, U8, U13, U14, U18 as part of the proposed internal and apical loops, but those of U10 and U21 as part of the proposed internal loop were found to be far less exposed than expected. Thus, our data are in better agreement with the proposed secondary structure of the riboswitch in complexes with aminoglycosides than with that of free RNA. For the riboswitch in complexes with neomycin B, ribostamycin, and paromomycin, we found highly similar CMC^+^‐modification patterns and excellent agreement with previous NMR studies. Differences between the chemical probing and MS data in the absence and presence of the aminoglycoside ligands were quantitative rather than qualitative (i. e., the same nucleobases were labeled, but to different extents) and can be rationalized by stabilization of both the proposed bulge and the apical loop by aminoglycoside binding. Our study shows that chemical probing and mass spectrometry can provide important structural information and complement other techniques such as NMR spectroscopy.

## Graphical abstract

**High‐resolution top‐down mass spectrometry** can directly localize and quantifiy nucleobase modifications from chemical probing reactions of RNA, providing important structural information. This study shows that high‐resolution mass spectrometry can be used for the localization and site‐specific, relative quantification of CMC+‐labeled RNA to within ∼5 %.

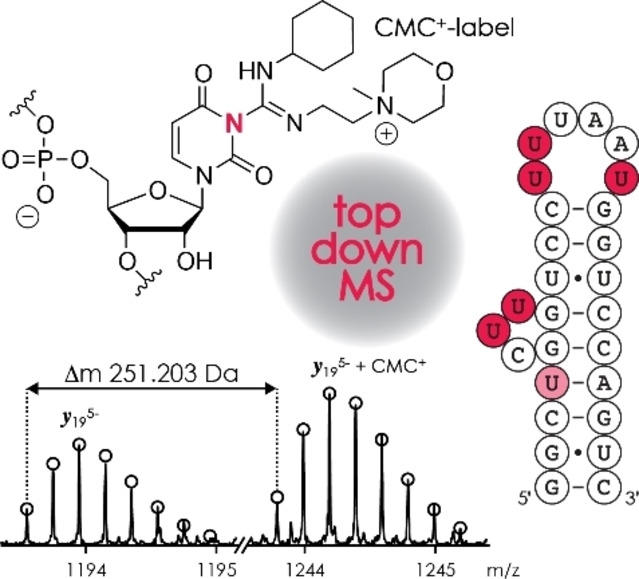

## Introduction

**Scheme 1:**
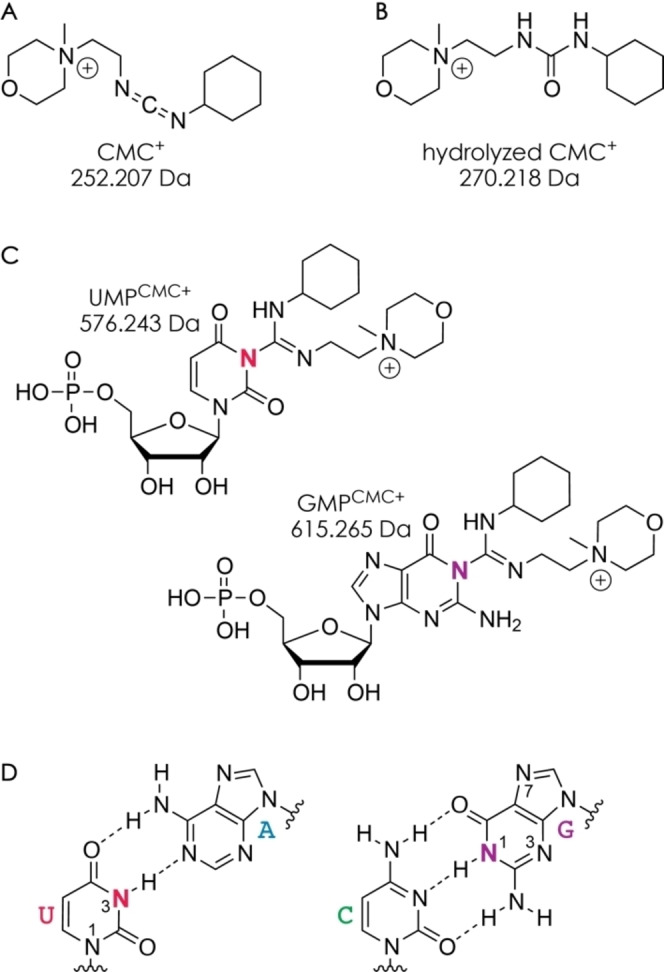
Chemical structures and calculated monoisotopic mass values of A) CMC^+^, B) hydrolyzed CMC^+^, and C) CMC^+^‐labeled UMP (UMP^CMC+^) and GMP (GMP^CMC+^); D) canonical Watson‐Crick base pairs in which N3 of U and N1 of G are protected from reaction with CMC^+^ shown in red and violet, respectively.

Mass spectrometry (MS) of ribonucleic acids (RNA) is a developing field with great potential for the characterization of RNA modifications and RNA‐ligand interactions.[^1^, ^2^, ^3^, ^4^, ^5^, ^6^, ^7^, ^8^, ^9^, ^10^, ^11^, ^12^, ^13^] For example, we have recently demonstrated that top‐down MS with collisionally activated dissociation (CAD) can be used for the label‐free, direct localization and relative quantification of the nucleobase methylations m^6^A, m^5^C, m^3^U, and m^5^U in RNA of up to 52 nt.^[11]^ Chemical probing reactions with the cationic carbodiimide CMC^+^ introduce another type of RNA nucleobase modification by reaction with N3 of uridine (U) and N1 of guanosine (G) (Scheme 1),[^14^, ^15^] the products of which are typically analyzed by resolving fragments from either RNA cleavage or primer extension with denaturing gel electrophoresis and estimating or individually quantifying band intensities.^[16]^ Inspired by studies in the Fabris group,[^17^, ^18^, ^19^, ^20^] we wondered if our CAD MS approach for nucleobase methylations would be equally useful for the label‐free, direct localization and relative quantification of CMC^+^‐modifications. Although complete sequence coverage by top‐down MS with CAD is limited to RNA of up to ∼100 nt as a result of secondary dissociation,[^21^, ^22^] we expected higher precision in the relative quantification of CMC^+^‐modifications (for nucleobase methylations, the standard deviation was ∼2 %)^[11]^ compared with reading out the chemical probing information from sequencing gels. As a major advantage, with top‐down MS, modified RNA can be analyzed directly, i. e., without primer extension, reverse transcription, and radioactive or fluorescent labels. Moreover, unlike primer extension, MS has no lower limit regarding RNA length, and is thus complementary to sequencing gels and newer methods based on capillary electrophoresis.[^23^, ^24^, ^25^, ^26^, ^27^, ^28^] In this study, we explore the potential of CAD MS for the direct localization and relative quantification of CMC^+^‐modified nucleobases of RNA by first studying mononucleotides and model hairpin RNA and then an artificial, neomycin‐sensing riboswitch aptamer domain^[29]^ in the absence and presence of aminoglycoside ligands.

## Results and Discussion

**Figure 1:**
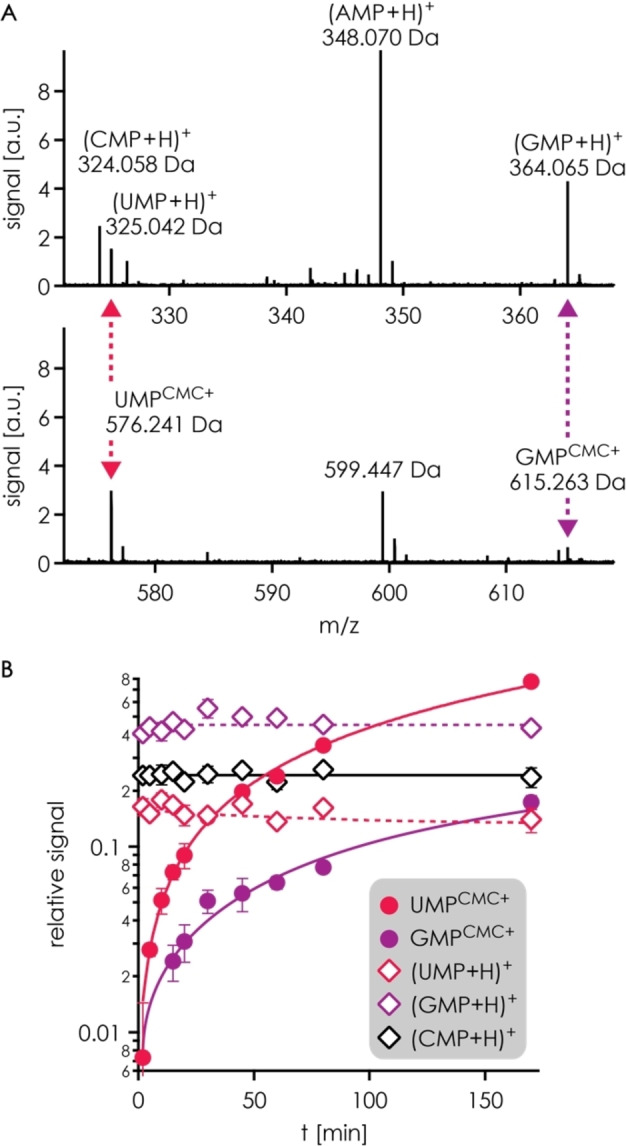
A) Sections of an ESI mass spectrum of a solution after 80 min reaction time show signals of protonated mononucleotides (top) and CMC^+^‐labeled UMP and GMP (bottom), the signal at 599.447 Da corresponds to a cluster of acetate and two hydrolyzed CMC^+^ ions (calculated monoisotopic mass: 599.449 Da); B) signals of CMC^+^‐labeled UMP and GMP and (UMP+H)^+^, (GMP+H)^+^, (CMP+H)^+^ relative to that of (AMP+H)^+^ versus reaction time (average values from 2–4 spectra for each time point, with error bars representing standard deviations) illustrates a higher reactivity of UMP in the reaction with CMC^+^ when compared to GMP; solid and dashed lines are meant to guide the eye.

To evaluate the reactivity of the different canonical RNA nucleobases in labeling reactions with the cationic carbodiimide CMC^+^ (C_14_H_26_N_3_O^+^, 252.207 Da, Scheme 1A), we have monitored the products from reaction of 5′‐phosphate mononucleotides of A, C, G, and U with CMC^+^ at pH 8.0 and room temperature by ESI MS. Following variable reaction times (0–170 min), the pH was lowered to 4.7 by 100‐fold dilution with 20 mM acetic acid and 20 mM ammonium acetate in H_2_O, resulting in complete hydrolysis of the unreacted carbodiimide within ∼20 min (Scheme 1B, Figure S1), after which the solutions were diluted again (see experimental section) and electrosprayed in positive ion mode (Figure 1A). Products from reaction with CMC^+^ were observed for UMP (calculated monoisotopic mass of UMP^CMC+^: 576.243 Da, Scheme 1C) and GMP (GMP^CMC+^: 615.265 Da) but not for CMP (CMP^CMC+^: 575.259 Da) and AMP (AMP^CMC+^: 599.270 Da; the signal at 599.447 Da in Figure 1A corresponds to a cluster composed of acetate and two hydrolyzed CMC^+^ ions), which confirms that CMC^+^ reacts with the nucleobases of U and G but neither with those of A and C nor the ribose or phosphate moieties.^[15]^ Consistent with this observation, the signal of (CMP+H)^+^ (calculated monoisotopic mass: 324.059 Da), relative to that of (AMP+H)^+^ (348.070 Da) – to account for differences in ion transmission and varying number of scans of the spectra – was not affected by reaction time (Figure 1B). By contrast, the signals of UMP^CMC+^ and GMP^CMC+^ (Scheme 1C), also relative to that of (AMP+H)^+^, increased from 0 at t=0 min to ∼0.77 and ∼0.17 at t=170 min, respectively. A corresponding decrease in the signals of (UMP+H)^+^ (325.043 Da) and (GMP+H)^+^ (364.065 Da) was, however, not observed. We attribute this finding to a far higher ionization efficiency of the CMC^+^‐labeled products with fixed positive charge (Scheme 1C) compared to that of the mononucleotides, which instead require nucleobase protonation for detection by ESI in positive ion mode. Although the MS data can thus be interpreted only in terms of relative reactivity, they clearly show that CMC^+^ reacts more readily with the nucleobase of U than that of G, in agreement with a ∼2‐fold higher equilibrium constant for the reaction between CMC^+^ and 5′‐phosphate mononucleotides of U compared to that with 5′‐phosphate mononucleotides of G.^[30]^

To investigate the use of ESI and CAD for the site‐specific, relative quantification of CMC^+^‐modifications, we conducted experiments with RNAs **1** and **2** (Table 1). Reaction times and temperatures were chosen such that only singly labeled RNA was formed, thereby avoiding possible changes in RNA structure due to multiple CMC^+^‐labels. Following variable reaction times and removal of reagent and buffer/salts by centrifugal concentration, RNA solutions were electrosprayed in negative ion mode. According to computational secondary structure prediction (http://rna.tbi.univie.ac.at//cgi‐bin/RNAWebSuite/RNAfold.cgi),^[31]^ RNAs **1** and **2** have the same stem with canonical Watson‐Crick base pairing (Scheme 1D) of the nucleobases 1–5 and 10–15, which protects N3 of U and N1 of G from reaction with CMC^+^, and differ only in their loop regions. Both loop motifs (RNA **1**: GCAA, RNA **2**: UUCG) are highly stable,[^32^, ^33^] with only the second nucleobase of UUCG being fully exposed to solvent[^34^, ^35^] and the proton of N1 of the first nucleobase of GCAA showing relatively slow exchange with solvent.^[36]^ We thus anticipated a higher reactivity of RNA **2** (UUCG‐loop) than RNA **1** (GCAA‐loop) in the labeling reaction with CMC^+^. Consistent with this prediction, ESI MS of a ∼1 : 1 mixture of RNA **1** and **2** after reaction with CMC^+^ for 90 min at room temperature indicated 0.4 % and 6.9 % labeled products, respectively (Table 1). The ratio in yield of CMC^+^‐labeled RNA **2** and **1** decreased from ∼18 (6.9 % to 0.4 %) at room temperature to ∼2 at 70 °C and 90 °C, which reflects the unfolding of both hairpin structures and the 2‐fold higher number of U in RNA **2** (4) compared to that of RNA **1** (2).

**Table 1:** Hairpin RNA studied, with loop regions highlighted in bold and nucleotides involved in Watson‐Crick base pairing underlined; * from 5′ to 3′ terminus, OH‐terminated; ^#^calculated by using the RNAfold web server as part of the ViennaRNA Package 2.0^[31]^ (http://rna.tbi.univie.ac.at/cgi‐bin/RNAWebSuite/RNAfold.cgi); yield of CMC^+^‐labeled RNA for different reaction temperatures and reaction times.

RNA	nt	sequence*	ΔG^#^	yield of CMC^+^‐labeled RNA			
				20 °C 90 min	50 °C 15 min	70 °C 6 min	90 °C 2 min
**1**	15	5′‐GAAGG **GCAA** CCUUCG‐3′	−30.5 kJ/mol	0.4 %	1.0 %	4.9 %	4.8 %
**2**	15	5′‐GAAGG **UUCG** CCUUCG‐3′	−24.7 kJ/mol	6.9 %	6.2 %	10.1 %	8.3 %

**Figure 2:**
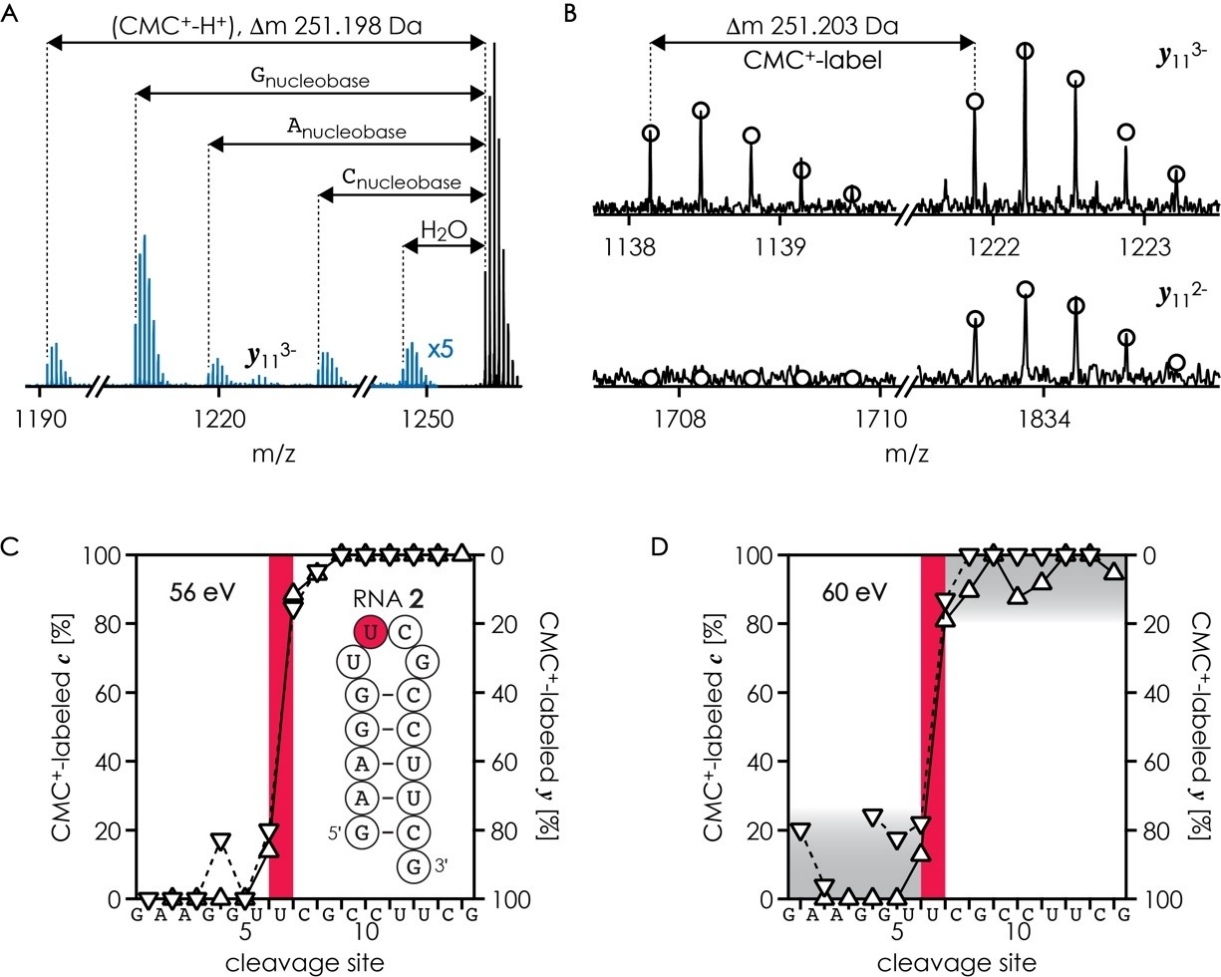
Sections of MS spectra illustrating A) small molecule losses and B) ***y***_11_ fragments with and without CMC^+^‐label from CAD of CMC^+^‐labeled ions of RNA **2** with a net charge of 4‐, (RNA **2** ^CMC+^‐5H)^4−^, at 60 eV laboratory frame energy, theoretical isotope distributions are shown as circles. Fractions of CMC^+^‐labeled ***c*** (upward triangles, left axis) and ***y*** (downward triangles, right axis) fragments from CAD of (RNA **2** ^CMC+^‐5H)^4−^ ions at C) 56 eV and D) 60 eV versus cleavage site; highlighted in red and gray are the preferred residue for CMC^+^‐labeling (U7) and the variation in fractions of CMC^+^‐labeled fragments at elevated energy, respectively.

Low‐energy CAD of CMC^+^‐labeled RNA **2** (from reaction at room temperature) with a net charge of −4, (RNA **2** ^CMC+^‐5H)^4−^, at 60 eV laboratory frame energy produced 15.1 % ***c*** and ***y*** fragments from phosphodiester backbone cleavage and 1.8 % ***a*** and ***w*** fragments (Table 2). Moreover, loss of deprotonated CMC^+^, (CMC^+^‐H^+^), with zero net charge (2.1 %), loss of unlabeled, uncharged nucleobases (from A, C, and G) (10.1 %), and loss of both (CMC^+^‐H^+^) and nucleobase (0.4 %) from (RNA **2** ^CMC+^‐5H)^4−^ ions was observed (Table 2, Figure 2A). Nucleobase loss and loss of deprotonated CMC^+^ (C_14_H_25_N_3_O, calculated mass 251.200 Da) decreased with decreasing energy used for CAD (Table 2), but at the expense of lower ***c*** and ***y*** fragment yields and correspondingly lower signal‐to‐noise ratio (S/N). Since CMC^+^ carries a fixed positive charge, deprotonated CMC^+^ must be a zwitterion with a net charge of zero. Therefore, the loss of (CMC^+^‐H^+^) from (RNA **2** ^CMC+^‐5H)^4−^ ions leading to (RNA **2**–**4**H)^4−^ ions (measured Δm 251.198 Da) must be accompanied by proton transfer from CMC^+^ to the RNA. Because CMC^+^ does not carry any acidic protons (Scheme 1A), this proton transfer reaction must be energetically demanding, in line with the relatively small percentage of ions showing loss of (CMC^+^‐H^+^) at all energies used (Table 2). Even less abundant were ions showing loss of both (CMC^+^‐H^+^) and nucleobase, which we attribute to preferential labeling of U and the relatively high stability of its glycosidic bond.^[4]^ Among all nucleobase losses in CAD of (RNA **2** ^CMC+^‐5H)^4−^ ions at 60 eV, that from G was most frequent (∼73 %), followed by C (∼16 %) and A (∼11 %); nucleobase loss from U was not observed in any of the spectra in this study.

**Table 2:** Percentage of (CMC−H) and nucleobase losses from CMC^+^‐labeled RNA **2** with a net charge of 4‐, (RNA **2** ^CMC+^‐5H)^4−^, and yield of ***c*** + ***y*** and ***a*** + ***w*** fragments in CAD of (RNA **2** ^CMC+^‐5H)^4−^ at different laboratory frame energies.

energy [eV]	energy/nt [eV]	(CMC^+^‐H^+^) loss	loss of (CMC^+^‐H^+^) and nucleobase	loss of unlabeled nucleobase	***c*** + ***y***	***a*** + ***w***
56	3.73	0.7 %	0.1 %	7.3 %	7.2 %	0.9 %
58	3.87	1.3 %	0.2 %	8.4 %	10.2 %	1.3 %
60	4.00	2.1 %	0.4 %	10.1 %	15.1 %	1.8 %

The loss of (CMC^+^‐H^+^) (Figure 2A) is detrimental to the relative quantification of site‐specific CMC^+^‐modifications as unlabeled ***c*** or ***y*** fragments cannot be distinguished from labeled ***c*** or ***y*** fragments which have lost (CMC^+^‐H^+^) during CAD. For example, 76 % of the ***y***_11_ fragments from CAD of (RNA **2** ^CMC+^‐5H)^4−^ ions at 60 eV laboratory frame energy showed labeling with CMC^+^ (Figure 2B), but since the complementary ***c***_4_ fragment did not show any labeling with CMC^+^, it is safe to assume that the 24 % ***y***_11_ fragments without CMC^+^ have lost their label during CAD. Moreover, ***c*** or ***y*** fragments showing nucleobase loss (from G) and no CMC^+^‐label could originate from dissociation of both labeled and unlabeled fragments. Since for RNA, nucleobase loss and phosphodiester backbone bond cleavage into ***c*** and ***y*** fragments are independent dissociation channels,[^4^, ^37^, ^38^] we have excluded fragments showing base loss from further analysis. In the absence of (CMC^+^‐H^+^) losses, phosphodiester backbone bond cleavage of CMC^+^‐labeled RNA should produce pairs of complementary ***c*** and ***y*** fragments with the CMC^+^‐label either on the ***c*** or the ***y*** fragment. Thus for each backbone cleavage site, the fractions of CMC^+^‐labeled ***c*** and ***y*** fragments should add to 100 % such that the data points for ***c*** and ***y*** fragments should lie on top of each other when plotted with reversed ordinate axes, similar to the fractions of methylated ***c*** and ***y*** fragments from CAD of RNA with *N* ^6^‐methyladenosine (m^6^ A), 5‐methylcytidine (m^5^ C), 3‐methyluridine (m^3^ U), and 5‐methyluridine (m^5^ U) modifications.^[11]^ In an alternative but equal representation, data points should fall on top of each other when fractions of CMC^+^‐labeled ***c*** and unlabeled ***y*** fragments are plotted on the same ordinate axis.^[11]^ Figure 2C shows the fractions of CMC^+^‐labeled ***c*** and ***y*** fragments from CAD of (RNA **2** ^CMC+^‐5H)^4−^ ions at 56 eV laboratory frame energy versus cleavage site, with values for ***c*** fragments plotted from 0 % to 100 % (left axis) and values for ***y*** fragments from 100 % to 0 % (right axis). The good agreement between the values for ***c*** and ***y*** fragments (Figure 2C) is consistent with little (CMC^+^‐H^+^) loss (i. e., in the majority of cases, the CMC^+^‐label can be found on either the ***c*** or the complementary ***y*** fragments).

For CAD of CMC^+^‐labeled RNA **2** at 56 eV, the fraction of CMC^+^‐labeled ***c*** fragments (Figure 2C, left axis) was zero for sites 1–5, and increased to ∼14 %, ∼88 %, and 100 % at sites 6, 7, and 9, respectively. The differences between fractions of CMC^+^‐labeled ***c*** fragments can be used to calculate the site‐specific extent of CMC^+^‐labeling of RNA **2**, that is, ∼14 % for U6, ∼74 % for U7, and ∼12 % for G9. In good agreement, the fractions of CMC^+^‐labeled ***y*** fragments (Figure 2C, right axis) indicate ∼20 % for U6, ∼65 % for U7, and ∼15 %. The high reactivity of U7 is consistent with it being fully exposed to solvent.[^34^, ^35^] Further, the data indicate a similar extent of CMC^+^‐labeling of U6 and G9. In the nuclear magnetic resonance (NMR) structures of UUCG loops, N1 of G (but not N3 of the first U) is involved in noncanonical hydrogen bonding between the first U and G.[^39^, ^40^] Our data thus suggest that the UUCG loop of RNA **2** is somewhat dynamic, and that the reactions of N1 of G9 and N3 of U6 with CMC^+^ are limited by solvent exposure rather than intrinsic nucleobase reactivity. At elevated energy (60 eV) used for CAD, larger disagreements between fractions of CMC^+^‐labeled ***c*** and ***y*** fragments were observed (∼10 % on average, Figure 2D), which we attribute to increased (CMC^+^‐H^+^) loss (Table 2). Since RNA **2** is predominantly labeled at U7, (CMC^+^‐H^+^) loss affects mainly ***c***_7_‐***c***_14_ and ***y***_9_‐***y***_14_. Nevertheless, the data in Figure 2D still indicate U7 as the preferred site for CMC^+^‐labeling.

**Figure 3:**
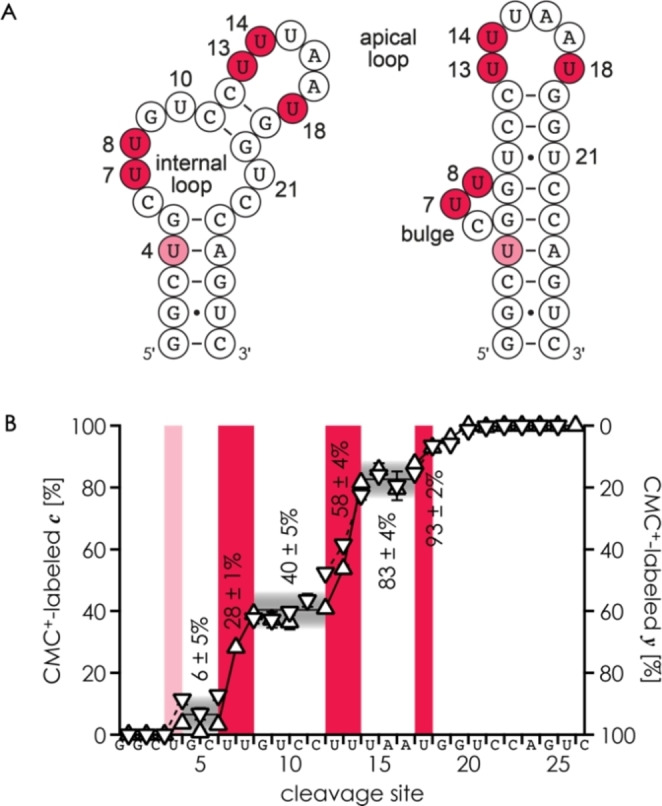
A) Secondary structures of free (left) and aminoglycoside‐bound (right) RNA **3** derived from NMR data.^[41]^ B) Fractions of CMC^+^‐labeled ***c*** (upward triangles, left axis) and ***y*** (downward triangles, right axis) fragments from CAD of (RNA **3** ^CMC+^‐8H)^7−^ ions of singly labeled RNA **3** (2 h reactions with CMC^+^) versus cleavage site (average values±standard deviation from experiments at 90.3 eV, 93.8 eV, and 97.3 eV); highlighted in red or pink are the CMC^+^‐labeled residues U4, U7, U8, U13, U14, and U18.

We then used our MS approach to study the structure of an artificial, neomycin‐sensing riboswitch aptamer domain^[29]^ (RNA **3**, 5′‐GGCUGCUUGUCCUUUAAUGGUCCAGUC‐3′) at room temperature. Reaction time and concentrations were chosen such that mostly singly labeled RNA was formed. For CAD experiments at three different energies (90.3 eV, 93.8 eV, and 97.3 eV), ions of singly labeled RNA with a net charge of 7‐, (RNA **3** ^CMC+^‐8H)^7−^, from ESI were isolated in the mass spectrometer prior to dissociation. The 15 nt (RNA **1** ^CMC+^‐5H)^4−^, 15 nt (RNA **2** ^CMC+^‐5H)^4−^, and 27 nt (RNA **3** ^CMC+^‐8H)^7−^ ions studied carry very similar charges per nucleotide (0.27, 0.27, 0.26, respectively), so that we can compare the energies used for CAD. For CAD of (RNA **1** ^CMC+^‐5H)^4−^ and (RNA **2** ^CMC+^‐5H)^4−^ ions, we used 56 eV, 58 eV, and 60 eV, which corresponds to 3.73 eV/nt, 3.87 eV/nt, and 4.00 eV/nt, respectively (Table 2). For CAD of the 27 nt (RNA **3** ^CMC+^‐8H)^7−^ ions, the energies per nucleotide were 3.34 eV/nt, 3.47 eV/nt, and 3.60 eV/nt, which is lower than the energies per nucleotide used for CAD of (RNA **1** ^CMC+^‐5H)^4−^ and (RNA **2** ^CMC+^‐5H)^4−^ ions. Accordingly, (CMC^+^‐H^+^) and nucleobase losses were lower in CAD of (RNA **3** ^CMC+^‐8H)^7−^ ions (Table 3) than in CAD of (RNA **2** ^CMC+^‐5H)^4−^ ions (Table 2), even though the yield of ***c*** and ***y*** fragments was higher. Figure S2 shows that the site‐specific fractions of CMC^+^‐labeled ***c*** and ***y*** fragments from CAD of (RNA **3** ^CMC+^‐8H)^7−^ ions of singly labeled RNA **3** at 90.3 eV, 93.8 eV, and 97.3 eV were virtually the same; average values with standard deviations are shown in Figure 3B.

**Table 3:** Percentage of (CMC−H) and nucleobase losses from CMC^+^‐labeled RNA **3** with a net charge of 7‐, (RNA **3** ^CMC+^‐8H)^7−^, and yield of ***c*** + ***y*** and ***a*** + ***w*** fragments in CAD of (RNA **3** ^CMC+^‐8H)^7−^ at different laboratory frame energies.

energy [eV]	energy/nt [eV]	(CMC^+^‐H^+^) loss	loss of (CMC^+^‐H^+^) and nucleobase	loss of unlabeled nucleobase	***c*** + ***y***	***a*** + ***w***
90.3	3.34	0.17 %	0.04 %	6.8 %	19.1 %	1.7 %
93.8	3.47	0.19 %	0.05 %	8.2 %	20.5 %	1.9 %
97.3	3.60	0.23 %	0.09 %	10.2 %	25.0 %	3.0 %

The CAD MS data for RNA **3** (Figure 3B) indicate reaction of CMC^+^ with U4, U7, U8, U13, U14, and U18, whereas U10, U15, U21, and U26 were, within error limits (up to 2.5 %), unreactive. Structures of RNA **3** in the absence of aminoglycoside ligands are not available, but NMR data suggest that the neomycin‐sensing riboswitch aptamer domain^[29]^ adopts a weak hairpin structure consisting of two helical stems separated by an internal loop (Figure 3A, left);^[41]^ the same secondary structure is predicted by computation.^[31]^ Moreover, the NMR study showed high imino proton exchange rates for U4, U10, U13, U14, U18 and U26 at 30 °C (with resonance broadening beyond detection for U4, U10, U13, and U18; rates for U7, U8, U15, and U21 were not reported),^[41]^ suggesting very high solvent exchange rates and a dynamic structure of the apical loop in the absence of aminoglycoside ligand. The site‐specific extent of CMC^+^‐modification at 20 °C calculated from the data in Figure 3B, U14 (25 %)≈U7 (22 %)>U13 (17 %)>U8 (12 %)≈U18 (11 %)>U4 (6 %), agrees well with high solvent exchange rates and is consistent with the predicted secondary structure in Figure 3A, except for U10, U15, and U21.

NMR solution structures of RNA **3** in complexes with aminoglycosides (paromomycin: pdb entry 2MXS, ribostamycin: 2 N0 J) at pH 6.2 were previously reported by Wöhnert and colleagues, along with dissociation constants (K_D_) of the complexes with neomycin B (10±2 nM), ribostamycin (330±30 nM), and paromomycin (5.13±0.26 μM) at pH 6.8.^[41]^ The secondary structures of RNA **3** in complexes with paromomycin and ribostamycin (Figure 3A, right) were the same, but the NMR data revealed differences in conformational dynamics with up to ∼7‐fold higher exchange rates (for U10 and U13) in the complexes with paromomycin.^[41]^ Complexes of RNA **3** with neomycin B are structurally and dynamically very similar to those with ribostamycin,^[41]^ although an NMR structure of the complex with neomycin B has not yet been published. Interestingly, our MS data for RNA **3** (Figure 3B), which show no evidence of a reaction of CMC^+^ with U10, U15, and U21, better agree with the secondary structure of the riboswitch complexes (Figure 3A, right) than that of the free RNA (Figure 3A, left). The corresponding NMR structures 2MXS and 2 N0 J show a wobble base pair between U10 and U21 that may also form in the free RNA but is too dynamic for detection. Highly dynamic base pairing between U10 and U21 (and U13 and U18) is also indicated by very broad imino proton signals observed in an earlier NMR study of free RNA **3** at 10 °C.^[42]^ However, the exact nature of the base pairing interactions is not yet clear, and could involve a dynamic equilibrium of different hydrogen bond patterns.[^43^, ^44^] Finally, our data suggest that intramolecular interactions protect the N3 atom of U15 of free RNA **3**, even though U15 is fully solvent‐exposed in the NMR structures 2MXS and 2 N0 J. Possible interactions of U15 are likely dynamic and include Wobble base pairing with G9 or hydrogen bonding to a phosphodiester moiety. Overall, our data for free RNA **3** which show high exposure to solvent of uridine nucleobases in the internal and apical loop regions (U7, U8, U13, U14, U18) and dynamic base pairing of U4 agree well with the NMR data, and add the additional information that U10 and U21 are less exposed than expected for a fully dynamic internal loop (Figure 3A).

In the next step, we probed the structure of RNA **3** in the presence of the aminoglycoside ligands neomycin B, ribostamycin, and paromomycin. With the dissociation constants reported in reference,^[41]^ we can calculate the percentage of 1 : 1 complexes for different concentrations of aminoglycoside ligands and a fixed concentration of RNA **3** (Table 4). For chemical probing of RNA‐aminoglycoside complex structures, we prepared solutions with 15 μM RNA **3** and 22.5 μM neomycin B, 45 μM ribostamycin, and 450 μM paromomycin, for which the percentage of 1 : 1 complexes should be 99.9 %, 98.9 %, and 99.9 %, respectively. We cannot exclude the possibility that our calculation overestimates the percentage of complexes in the pH 8 solution used for labeling with CMC^+^ since the dissociation constants were determined for solutions at pH 6.8, but even for 10‐fold higher K_D_ values, the percentage of complexes would still be 99 %, 91 %, and 90 % for neomycin B, ribostamycin, and paromomycin, respectively.

**Table 4:** Percentage of 1 : 1 complexes calculated for RNA **3** (15 μM) and different concentrations of the aminoglycosides neomycin B, ribostamycin, and paromomycin calculated from K_D_ values from reference.^[41]^

aminoglycoside	K_D_	22.5 μM ligand (1 : 1.5)	45 μM ligand (1 : 3)	450 μM ligand (1 : 30)
neomycin B	10±2 nM	**99.9 %**	100.0 %	100.0 %
ribostamycin	330±30 nM	96.1 %	**98.9 %**	99.9 %
paromomycin	5.13±0.26 μM	70.0 %	86.2 %	**98.8 %**

**Figure 4:**
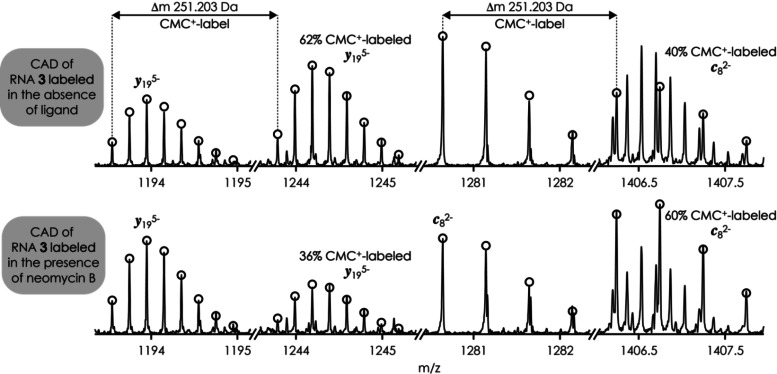
Segments of MS spectra from CAD (93.8 eV laboratory frame energy) of (RNA **3** ^CMC+^‐8H)^7−^ ions of singly labeled RNA **3** from 2 h reactions with CMC^+^ in the presence (bottom) and absence (top) of neomycin B illustrating ***c***_8_^2−^ and ***y***_19_^5−^ fragments with and without CMC^+^‐label; theoretical isotope distributions of these fragments are shown as circles.

The CAD spectra of singly labeled RNA **3** from reaction with CMC^+^ in the presence and absence of aminoglycoside ligands showed clear differences, as illustrated in Figure 4 for neomycin B and fragments from phosphodiester backbone cleavage at site 8. The complementary fragments ***c***_8_^2−^ and ***y***_19_^5−^ showed 40 % and 62 % CMC^+^ labeling in the absence of ligand, respectively, and 60 % and 36 % CMC^+^ labeling in the presence of neomycin B. Including the contribution of the far less abundant ***c***_8_^3−^ and ***y***_19_^4−^ fragments (not shown), the overall fractions of labeled ***c***_8_ and ***y***_19_ were 39 % and 61 % (adding up to 100 %) in the absence of ligand, respectively, and 58 % and 33 % in the presence of neomycin B (adding up to 91 %).

**Figure 5:**
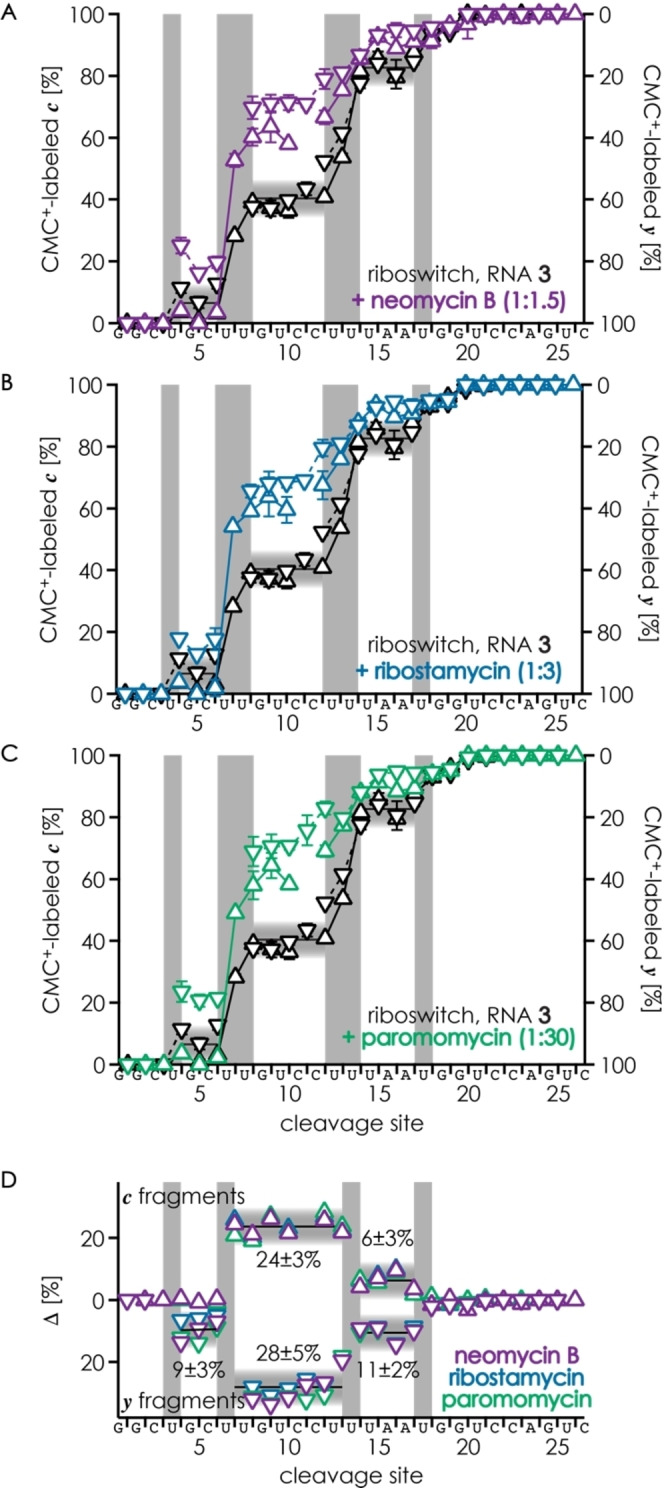
Fractions of CMC^+^‐labeled ***c*** (upward triangles, left axis) and ***y*** (downward triangles, right axis) fragments from CAD of (RNA **3** ^CMC+^‐8H)^7−^ ions of singly labeled RNA **3** from 2 h reactions with CMC^+^ in the presence of aminoglycoside versus cleavage site (A–C) and Δ% values illustrating the differences between %‐values for complexes and free RNA with data for ***c*** (upward triangles) and ***y*** (downward triangles) fragments plotted above and below 0, respectively (D); highlighted in gray are the CMC^+^‐labeled residues U4, U7, U8, U13, U14, and U18.

Figure 5A–C shows the fractions of all CMC^+^‐labeled ***c*** and ***y*** fragments from CAD of singly labeled RNA **3** from reaction with CMC^+^ in the presence of neomycin B, paromomycin, and ribostamycin, respectively, along with the data for free RNA **3** from Figure 3B for comparison. Consistent with a virtually identical average structure of RNA **3** in complexes with all three aminoglycosides,^[41]^ the data for all aminoglycosides are very similar, which becomes even clearer when plotting the differences between %‐values for free and bound RNA (Figure 5D). Strikingly, for the reactions of RNA **3** with CMC^+^ in the presence of aminoglycosides, the data points for ***c*** and ***y*** fragments at sites 4–12 show relatively large gaps instead of lying on top of each other (Figure 5A–C). Although such gaps were also observed for free RNA **3**, they were less frequent and virtually absent for sites 8–10 (Figure 3B). We noticed that (CMC^+^‐H^+^) loss from (RNA **3** ^CMC+^‐8H)^7−^ ions was higher when RNA **3** was reacted with CMC^+^ in the presence of aminoglycosides (e. g., 0.31 % for neomycin B compared to 0.19 % in the absence of aminoglycoside ligands, both at 93.8 eV, Table 3), and conclude that the stability of the CMC^+^‐label in the gas phase depends somewhat on the position of the label within the RNA sequence. Nevertheless, the data in Figure 5 clearly show that for all aminoglycosides studied, the fraction of CMC^+^‐labeled ***c*** fragments increased by ∼24 % at site 7 (from ∼40 % for free RNA to ∼64 % for RNA in the presence of aminoglycosides), along with a corresponding decrease at sites 14 and 18 (Figure 5D). The data for ***y*** fragments show a similar increase of CMC^+^‐labeling at site 7 (∼28 %) and a smaller increase at site 4 (∼9 %), the latter of which was, however, not observed for ***c*** fragments (Figure 5D). The data in Figure 5 thus suggest that aminoglycoside binding increases the exposure of U7 and decreases the exposure of U14 and U18, which can be rationalized by stabilization of the bulge (in which U7 is turned outward) and the apical loop (in which N3 of U14 is protected by hydrogen bonding to the phosphodiester moiety of A17 and the N3 atoms of U13 and U18 are protected by non‐canonical[^45^, ^46^] base pairing)^[41]^ by aminoglycoside binding. No change in reactivity was observed for U8, U10, U15, U21, and U26 (Figure 5D), of which only U8 showed significant reactivity (∼12 %) in the free RNA **3**. Apparently, the exposure to solvent of U8 is not affected by aminoglycoside binding. Finally, our data did not reflect the up to ∼7‐fold higher exchange rates of U10 and U13 in the complexes with paromomycin when compared to ribostamycin.

The fractions of CMC^+^‐labeled fragments in the presence and absence of aminoglycoside ligands (Figure 5A–C) are, however, not directly comparable since the yield of CMC^+^‐labeled RNA **3** was different in the presence and absence of aminoglycoside ligands (Table 5). Moreover, the labeling reaction of RNA with CMC^+^ is reversible,^[15]^ such that loss of CMC^+^ can occur during sample cleanup and storage, and even in the basic (pH ∼10) solution used for electrospray ionization. For example, after removal of small molecules and ions (CMC^+^, buffer, salts, and ligands) using centrifugal concentrators, the ESI spectra indicated 32 % singly labeled RNA **3** for the reaction in the absence of aminoglycoside ligands, and 20–23 % for the reactions in the presence of aminoglycoside ligands (Table 5). These samples were immediately cooled on ice and stored at −20 °C for 4 weeks before the CAD experiments (Figures 3 and 5) were performed. The ESI spectra of the same samples after 4 weeks showed 27 % singly labeled RNA **3** for the sample from reaction in the absence of aminoglycoside ligands, and 13–16 % for the samples from reaction in the presence of aminoglycoside ligands (Table 5). For a quantitative comparison of the extent of site‐specific labeling of RNA in the absence and presence of different ligands, the label would have to be fully stable in solution as well as during CAD. However, because the yield of labeled RNA **3** was consistently lower in the presence of aminoglycoside ligands, the above conclusions from the data in Figure 5 are still valid.

**Table 5:** Percentage of unlabeled and labeled RNA **3** indicated by ESI MS directly after removal of small molecules and ions by centrifugal concentration and after subsequent sample storage at −20 °C for 4 weeks; the reactions with CMC^+^ used RNA **3** (15 μM) in the absence or presence of neomycin B (22.5 μM), ribostamycin (45 μM), or paromomycin (450 μM).

ligand	after removal of small molecules & ions			after storage at −20 °C for 4 weeks		
	0 CMC^+^	1 CMC^+^	2 CMC^+^	0 CMC^+^	1 CMC^+^	2 CMC^+^
no ligand	61 %	32 %	7 %	68 %	27 %	5 %
neomycin B	75 %	23 %	2 %	83 %	16 %	1 %
ribostamycin	77 %	21 %	2 %	86 %	13 %	1 %
paromomycin	79 %	20 %	1 %	85 %	14 %	1 %

## Conclusions

In this study, we have shown that high‐resolution mass spectrometry can be used for the localization and site‐specific, relative quantification of CMC^+^‐labeled RNA to within ∼5 %. The CAD MS data for the neomycin‐sensing riboswitch generally agree well with NMR data reported in the literature, and show that mass spectrometry is a promising technique for chemical probing of smaller RNA, especially when RNA dynamics complicate interpretation of NMR data or interfere with RNA crystallization. In future experiments, we will explore more stable chemical labels for structural probing and extend the approach to larger RNA.

## Experimental

*N*‐Cyclohexyl‐*N*′‐*β*‐(4‐methylmorpholinium)ethylcarbodiimide *p*‐toluenesulfonate (CMCT), 5′‐phosphate mononucleotides of A (adenosine), C (cytidine), G (guanosine), and U (uridine), piperidine, imidazole, acetic acid, ammonium acetate, ammonium bicarbonate, magnesium citrate, and sulfate salts of neomycin B (C_23_H_46_N_6_O_13_ ⋅ xH_2_SO_4_), ribostamycin (C_17_H_34_N_4_O_10_ ⋅ xH_2_SO_4_), and paromomycin (C_23_H_45_N_5_O_14_ ⋅ xH_2_SO_4_,) were purchased from Sigma‐Aldrich (Vienna, Austria). H_2_O was purified to 18 MΩ⋅cm at room temperature using a Milli‐Q system (Millipore, Austria) and CH_3_OH (VWR, Austria) was HPLC‐grade. Hairpin RNAs **1** and **2** were prepared by solid phase synthesis, purified by HPLC, and desalted by diluting ∼100 μl RNA solution in H_2_O with ∼400 μl aqueous ammonium acetate (100 mM) solution, followed by concentration to ∼100 μl using centrifugal concentrators (Vivaspin 500, MWCO 3000, Sartorius AG, Germany); the concentration‐dilution process was repeated 5 times and followed by 6 cycles of concentration and dilution with H_2_O. RNA **3** was purchased from Dharmacon (USA), purified by HPLC, and desalted as described above. RNA concentration was determined by UV absorption at 260 nm using an Implen Nano PhotometerTM (Implen, Germany).

To monitor the reactions of CMC^+^ with mononucleotides, CMCT (1 mM) and 5′‐phosphate mononucleotides of A, C, G, and U (300 μM each) were dissolved in H_2_O with 20 mM ammonium acetate and 12 mM ammonium bicarbonate at pH 8.0. After variable reaction times (0–170 min), reaction quenching was initiated by lowering the pH to 4.7 (100‐fold dilution with 20 mM acetic acid and 20 mM ammonium acetate in H_2_O). After another 25 min to allow for complete hydrolysis of CMC^+^, the solutions were diluted (1 : 1) in H_2_O/CH_3_OH (1 : 1) with 20 mM ammonium bicarbonate and electrosprayed in positive ion mode. The final composition of the solution for ESI was 1.5 μM per mononucleotide in 3 : 1 H_2_O/CH_3_OH with 10 mM acetic acid, 10 mM ammonium acetate, and 10 mM ammonium bicarbonate as well as unreacted, hydrolyzed CMC^+^ (up to 5 μM).

For CMC^+^‐labeling of hairpin RNAs, aqueous solutions of RNA **1** and **2** (10 μM each) with CMCT were incubated for 90 min at 20 °C, 15 min at 50 °C, 6 min at 70 °C, or 2 min at 90 °C. The CMCT concentration for the reaction at 20 °C was 4 mM, and the pH adjusted to ∼8 with 15 mM ammonium acetate and 6 mM ammonium bicarbonate. For the reactions at 50 °C, 70 °C, and 90 °C, the CMCT concentration was 6 mM, and ammonium acetate and ammonium bicarbonate concentrations were 41 mM and 9 mM, respectively. Reactions were stopped by removing CMCT using the desalting procedure described above, and the RNA (1 μM each) was electrosprayed in negative ion mode from solutions in 1 : 1 H_2_O/CH_3_OH with 10 mM piperidine and 20–25 mM imidazole as additives.

For CMC^+^‐labeling of the riboswitch aptamer domain, 15 μM RNA **3** and 6 mM CMCT were incubated in H_2_O with 41 mM ammonium acetate, 9 mM ammonium bicarbonate, and 0.1 mM magnesium citrate at 20 °C for 120 min in the absence or presence of aminoglycoside ligands (neomycin B: 22.5 μM, ribostamycin: 45 μM, paromomycin: 450 μM). Reactions were stopped by removing CMCT using the desalting procedure described above, and the RNA (1 μM) was electrosprayed in negative ion mode from solutions in 1 : 1 H_2_O/CH_3_OH with 2 mM piperidine and 9 mM imidazole as additives.

All MS experiments were performed on a 7 T Fourier transform ion cyclotron resonance (FT‐ICR) instrument (Apex Ultra, Bruker, Austria) equipped with an ESI source, a linear quadrupole for ion isolation, and a collision cell for CAD. ESI spectra were obtained by operation of the linear quadrupole in transmission (radiofrequency‐only) mode. For CAD, ions of interest were isolated in the quadrupole and dissociated in the collision cell using the laboratory frame collision energy indicated. Data reduction utilized the SNAP2 algorithm (Bruker, Austria) or FAST MS, a software programmed by the first author, as well as manual inspection of the spectra.

## Conflict of interest

The authors declare no conflict of interest.

1

## Supporting information

As a service to our authors and readers, this journal provides supporting information supplied by the authors. Such materials are peer reviewed and may be re‐organized for online delivery, but are not copy‐edited or typeset. Technical support issues arising from supporting information (other than missing files) should be addressed to the authors.

Supporting Information

## Data Availability

The data that support the findings of this study are available from the corresponding author upon reasonable request.
