# Supplementary material for: RNA Chemical Labeling with Site‐Specific, Relative Quantification by Mass Spectrometry for the Structural Study of a Neomycin‐Sensing Riboswitch Aptamer Domain
Source: Chempluschem. 2022 Oct 11;87(11):e202200256. doi: 10.1002/cplu.202200256 (PMC9828840; doi:10.1002/cplu.202200256)
Supplement: Supplementary file 1 — Supporting Information [file CPLU-87-0-s001.pdf]

ChemPlusChem

Supporting Information

RNA Chemical Labeling with Site-Specific, Relative Quantification by Mass Spectrometry for the Structural Study of a Neomycin-Sensing Riboswitch Aptamer Domain

Michael Palasser and Kathrin Breuker*

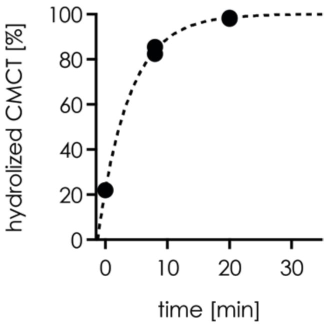

Figure S1: Percentage of hydrolyzed CMCT versus time after lowering the solution pH to 4.7 by addition of acetic acid (15 mM) and ammonium acetate (20 mM) at room temperature.

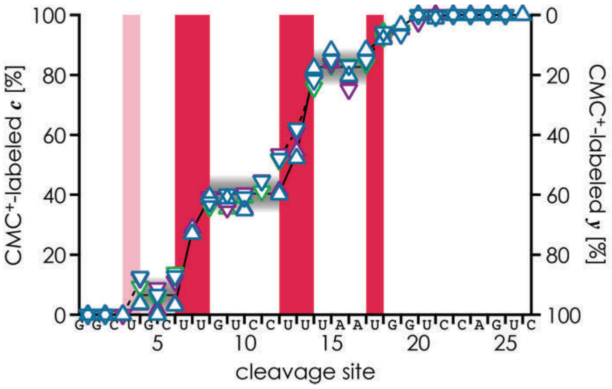

Figure S2: Fractions of CMC⁺-labeled ***c*** (upward triangles, left axis) and ***y*** (downward triangles, right axis) fragments from CAD of (RNA **3**$^{CMC+}$-8H)$^{7-}$ ions of singly labeled RNA **3** (2 h reactions with CMC⁺) versus cleavage site from experiments at 90.3 eV (blue), 93.8 eV (violet), and 97.3 eV (green); highlighted in red or pink are the CMC⁺-labeled residues U4, U7, U8, U13, U14, and U18.
